# Psoriatic arthritis is associated with adverse body composition predictive of greater coronary heart disease and type 2 diabetes propensity – a cross-sectional study

**DOI:** 10.1093/rheumatology/keaa604

**Published:** 2020-11-04

**Authors:** Lyn D Ferguson, Jennifer Linge, Olof Dahlqvist Leinhard, Rosemary Woodward, Pauline Hall Barrientos, Giles Roditi, Aleksandra Radjenovic, Iain B McInnes, Stefan Siebert, Naveed Sattar

**Affiliations:** 1 Institute of Cardiovascular and Medical Sciences, University of Glasgow, Glasgow, UK; 2 AMRA Medical, Linköping, Sweden; 3 Department of Health, Medicine and Caring Sciences, Linköping University, Linköping, Sweden; 4 Glasgow Clinical Research Imaging Facility, Queen Elizabeth University Hospital, Glasgow, UK; 5 Institute of Infection, Immunity, and Inflammation, University of Glasgow, Glasgow, UK

**Keywords:** psoriatic arthritis, ectopic fat, obesity, diabetes, CHD

## Abstract

**Objectives:**

To compare body composition in PsA with metabolic disease free (MDF) controls and type 2 diabetes and assess body-composition predicted propensity for cardiometabolic disease.

**Methods:**

Detailed MRI body composition profiles of 26 PsA participants from the IMAPA study were compared with 130 age, sex and BMI-matched MDF controls and 454 individuals with type 2 diabetes from UK Biobank. The body-composition predicted propensity for coronary heart disease (CHD) and type 2 diabetes was compared between PsA and matched MDF controls.

**Results:**

PsA participants had a significantly greater visceral adipose tissue (VAT) volume [mean 5.89 l (s.d. 2.10 l)] compared with matched-MDF controls [mean 4.34 l (s.d. 1.83 l)] (*P* <0.001) and liver fat percentage [median 8.88% (interquartile range 4.42–13.18%)] compared with MDF controls [3.29% (1.98–7.25%)] (*P* <0.001). These differences remained significant after adjustment for age, sex and BMI. There were no statistically significant differences in VAT, liver fat or muscle fat infiltration (MFI) between PsA and type 2 diabetes. PsA participants had a lower thigh muscle volume than MDF controls and those with type 2 diabetes. Body composition-predicted propensity for CHD and type 2 diabetes was 1.27 and 1.83 times higher, respectively, for PsA compared with matched-MDF controls.

**Conclusion:**

Individuals with PsA have an adverse body composition phenotype with greater visceral and ectopic liver fat and lower thigh muscle volume than matched MDF controls. Body fat distribution in PsA is more in keeping with the pattern observed in type 2 diabetes and is associated with greater propensity to cardiometabolic disease. These data support the need for greater emphasis on weight loss in PsA management to lessen CHD and type 2 diabetes risk.

**Rheumatology key messages**

- PsA patients have greater visceral and ectopic liver fat, and lower thigh-muscle volume, than metabolic-disease- free controls.
- This adverse body fat distribution is associated with greater propensity to type 2 diabetes and CHD.
- This study supports the need for weight loss interventions in PsA to lessen cardiometabolic risk.

## Introduction

Increased BMI is strongly associated with PsA [1]. However, BMI is a global adiposity marker, and does not capture regional fat. Central obesity, assessed increased waist circumference, is independently associated with higher odds of psoriasis [2]. The site of fat storage is important as visceral adipose tissue (VAT) and ectopic fat including fat in the liver and skeletal muscle are associated with increased metabolic risk [3, 4]. 

Body composition profiling with MRI allows detailed assessment of body fat distribution including quantification of abdominal subcutaneous adipose tissue (ASAT), VAT, ectopic fat including liver fat and muscle fat infiltration (MFI), as well as fat-free thigh muscle volume (FFMV). Higher VAT and MFI have been associated with greater coronary heart disease (CHD) and type 2 diabetes prevalence, and higher liver fat with type 2 diabetes, independently of BMI [5]. Greater MFI and lower FFMV may assist in the diagnosis of sarcopenia [6], for which there are currently few data available in PsA.

We aimed to characterize for the first time the detailed body composition profile of PsA using MRI compared with age, sex and BMI-matched metabolic disease free (MDF) controls and type 2 diabetes, and to relate body composition to propensity for CHD and type 2 diabetes in PsA *vs* MDF controls. 

## Methods

### Study design and participants

PsA participants were recruited from the Immune Metabolic Associations in PsA (IMAPA) study. This open label mechanistic study investigated cardiometabolic outcomes in psoriatic disease treated with apremilast [7]. PsA participants were required to fulfill the Classification for PsA (CASPAR) criteria [8] and Scottish Medicines Consortium guidelines for apremilast [9]. Exclusion criteria included other autoimmune rheumatic diseases, severe renal disease, transaminitis > four times upper limit of normal, significant recent weight loss, pregnancy, insulin-dependent diabetes, and biologic/leflunomide treatment. Between June 2017 and November 2019, 29 PsA participants underwent body composition profiling with a 3.0-T MRI scanner (Prisma, Siemens, Erlangen, Germany) at baseline prior to starting apremilast. After excluding three participants with type 2 diabetes, body composition data from 26 PsA participants were compared with 130 age, sex and BMI-matched MDF controls from UK Biobank [10], a large, general population-based cohort of 502 682 participants aged 40–70 years, which included MR imaging of 10 000 individuals using a 1.5-T MRI scanner (Aera, Siemens, Erlangen, Germany). MDF was defined as per Linge et al. [5] and excluded individuals with cardiovascular disease, diabetes, liver disease, respiratory, gastrointestinal, and neurological conditions, cancers, RA, PsA, and additionally psoriasis. Healthy controls were matched 1:5, first on sex and then by choosing the nearest MDF participants to each case using age (±5years) and BMI (±2kg/m^2^). Distance was calculated as the Euclidean distance and matching was done without replacement. PsA imaging data was also compared with UK Biobank participants with type 2 diabetes (*n* = 454), defined as self-report of diabetes diagnosed by a doctor and age at diagnosis ≥30 years.

### Outcomes

A body composition profile (BCP) was defined as a combination of variables that together described the fat and/or muscle distribution of a group and included VAT volume (l); VATindex (VAT normalized by height squared); ASAT volume (L); ASATindex (ASAT normalized by height squared); liver fat (%); MFI (%); thigh fat-free muscle volume (FFMV); FFMV corrected for height and compared with a sex- and BMI-matched virtual control group (FFMV_VCG_) (unit: number of s.d.s from mean value of VCG); and weight-to-muscle-ratio (kg/l). These data, or derivations, were then plotted in six-axis radar charts (body composition profile plots) as described previously [5]. All participants underwent standardized imaging protocols with analyses read in a blinded fashion. Between MR scanner bias and reproducibility coefficients have been recently published by AMRA Medical AB, Linkoping, Sweden [11]. The propensity for CHD or type 2 diabetes based on body composition was calculated according to the method described by Linge et al. [12]. For each PsA participant, the propensity for these conditions was estimated by the prevalence of CHD and type 2 diabetes within personalized control groups from UK Biobank matched for sex and body fat distribution (VAT index, ASAT index, liver fat and MFI) by applying the adaptive k-nearest neighbours algorithm.

### Statistical analysis

All analyses were performed using R version 3.4.4 (The R Foundation, Vienna, Austria). Continuous data presented as mean (s.d.), except liver fat presented as median and interquartile range (IQR); categorical data presented as number (*n*) and percentage (%). Body composition in PsA was compared with age, sex and BMI-matched MDF controls using mixed effects linear regression and to type 2 diabetes using linear regression. Analyses were further adjusted for age, sex and BMI. The difference in distributions of variables used for matching (age and BMI) between PsA and matched MDF controls were tested using Wilcoxon signed-rank test. Body-composition predicted CHD and type 2 diabetes propensities were compared between PsA and MDF controls using Wilcoxon signed-rank test.

The IMAPA study received ethical approval from West of Scotland Research Ethics Committee 4 (Reference 17/WS/0006). The UK Biobank study was approved by the North West Multicentre Ethics Research Committee (Application Number 6569). All participants provided written informed consent for data collection and analysis.

## Results

PsA participants were well matched for age and sex with MDF controls but were younger and included a greater proportion of females compared with type 2 diabetes participants (Table 1). There was no statistically significant difference in BMI between PsA, MDF controls and type 2 diabetes. Detailed characteristics of PsA participants including baseline disease activity and cardiometabolic parameters are outlined in supplementary Table S1, available at *Rheumatology* online.

**Table 1:** Comparison of body composition parameters between PsA, matched metabolic disease free (MDF) controls, and type 2 diabetes

Variable	PsA (*n* = 26)	MDF controls (*n* = 130)	*P*-value^a^	Adj. *P* value^b^	Type 2 diabetes (*n* = 454)	*P*-value^c^	Adj. *P* value^d^
Age (years)	56.0 (9.0)	57.4 (6.5)	0.766	—	65.4 (6.9)	< 0.001	—
Female (*n*, %)	13 (50)	65 (50)	1.000	—	158 (34.8)	<0.001	—
BMI (kg/m^2^)	31.2 (6.4)	30.5 (5.3)	0.799	—	29.9 (5.2)	0.397	—
VAT (l)	5.89 (2.10)	4.34 (1.83)	<0.001	<0.001	5.93 (2.56)	0.937	0.434
Visceral fat index (l/m^2^)	2.06 (0.73)	1.52 (0.64)	<0.001	<0.001	2.03 (0.84)	0.842	0.243
ASAT (l)	10.48 (4.90)	9.42 (4.86)	0.002	0.063	8.58 (3.93)	0.019	0.318
Abdominal fat index (l/m^2^)	5.87 (2.39)	4.93 (2.29)	<0.001	<0.001	5.04 (1.92)	0.036	0.017
Liver fat (%)	8.88 (4.42–13.18)	3.29 (1.98–7.25)	<0.001	<0.001	6.13 (2.77–11.63)	0.160	0.999
MFI (%)	7.74 (2.57)	7.43 (1.95)	0.316	0.283	8.61 (2.29)	0.062	0.165
FFMV, l	10.40 (2.38)	11.43 (2.88)	0.001	<0.001	11.1 (2.34)	0.142	0.002
FFMV_VCG_, s.d._VCG_	−0.74 (1.71)	0.40 (1.14)	<0.001	<0.001	−0.31 (1.03)	0.052	<0.001
WMR (kg/l)	8.89 (1.95)	7.96 (1.99)	<0.001	<0.001	8.03 (1.52)	0.006	<0.001

Values are mean (s.d.). For liver fat median (interquartile range).

a PsA *vs* MDF controls.

b PsA *vs* MDF controls adjusted for age, sex, and BMI.

c PsA *vs* Type 2 diabetes.

d PsA *vs* Type 2 diabetes adjusted for age, sex, and BMI.

ASAT: abdominal subcutaneous adipose tissue; FFMV: fat-free muscle volume; FFMV_VCG_: virtual control group (VCG) adjusted FFMV; MFI: muscle fat infiltration; VAT: visceral adipose tissue; WMR: weight-to-muscle ratio.

**Fig. 1:**
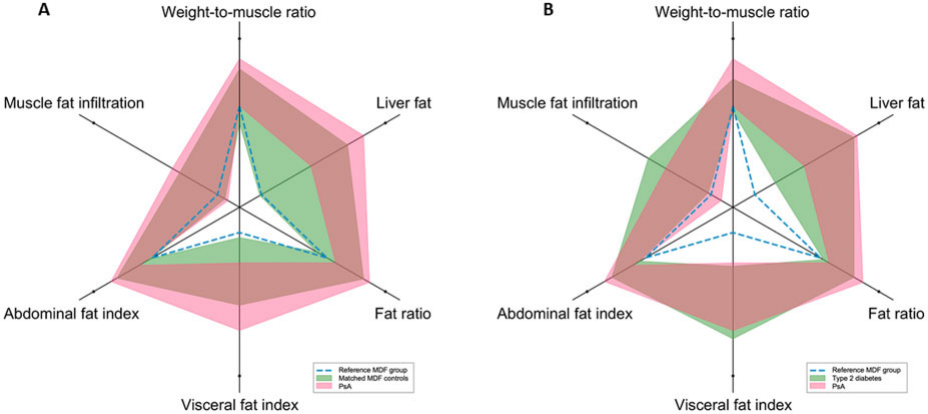
Body composition profiles of PsA compared with (**A**) matched metabolic-disease-free (MDF) controls, and (**B**) type 2 diabetes Pink and green fields represent the interquartile ranges of PsA cases (pink) and either matched-MDF controls (**A**) or type 2 diabetes (**B**) (green), brown areas the overlap between groups, and dashed blue lines the median of an unmatched MDF reference group.

PsA participants had a significantly greater VAT volume [mean 5.89 l (s.d. 2.10 l)] compared with matched MDF controls [4.34 L (s.d. 1.83 l)] (*P* <0.001), and liver fat percentage [median 8.88% (IQR 4.42–13.18%)] compared with matched MDF controls [3.29% (1.98–7.25%)] (*P* <0.001) (Table 1, Fig. 1A). These differences persisted after adjustment for age, sex and BMI. There were no statistically significant differences in VAT, liver fat or MFI between PsA and type 2 diabetes (Table 1, Fig. 1B). PsA participants had lower FFMV and FFMV_VCG_, and greater weight-to-muscle-ratio than MDF controls and type 2 diabetes participants after adjustment (Table 1), indicating lower thigh muscle volume in PsA (Table 1).

The distribution of body-composition predicted propensity for CHD and type 2 diabetes for PsA participants (red) in relation to the complete UK Biobank dataset (grey) is outlined in supplementary Fig. S1, available at *Rheumatology* online, together with mean disease propensities. PsA participants had 1.27 times higher mean CHD propensity and 1.83 times higher mean type 2 diabetes propensity compared with their matched MDF controls suggestive of a body composition phenotype with stronger association to type 2 diabetes.

## Discussion

This is the first study to demonstrate PsA has an adverse body fat distribution compared with healthy controls and which is more in line with type 2 diabetes on MRI with evidence of elevations in VAT and liver fat. Such findings, in turn, predict greater CHD and type 2 diabetes propensity, by 27% and 83% respectively, compared with MDF controls. PsA participants also had lower thigh muscle volume than MDF controls and type 2 diabetes participants.

Previous studies showed greater mean total body fat in PsA (46% ± s.d. 5.7%) compared with healthy controls (43.4% ± 5.5%) (*P* =0.04); however, individual fat compartments were not measured [13]. One prior study using CT has reported greater visceral fat in psoriasis compared with age- and sex-matched controls [14], but there was no data on liver fat. Our finding of higher liver fat fraction in PsA fits with increased Non-Alcoholic Fatty Liver Disease (NAFLD) prevalence observed in psoriatic disease [15], linked in turn to hepatic insulin resistance [4] and over twice the risk of diabetes compared with those without NAFLD [16].

Of particular note, the reported body composition in PsA is more strongly associated with type 2 diabetes, and to a lesser degree CHD, compared with age, sex and BMI-matched healthy individuals, consistent with observational data [17]. Recent genetic studies have suggested there is a causal link between adverse body fat distribution and cardiometabolic outcomes [18], suggesting that the PsA body composition may be causally linked to such risks.

PsA participants also demonstrated lower thigh fat-free muscle volume (FFMV) compared with MDF controls. Previous work has shown an association between lower FFMV with functional measures of low muscle strength and poor physical performance characteristic of sarcopenia [6]. There are little data on muscle volume in PsA; however, a previous study demonstrated lower skeletal muscle index and higher sarcopenia incidence in PsA compared with controls without inflammatory joint disease [19]. This may relate to decreased physical activity secondary to joint pain and/or underlying chronic inflammation.

Study strengths include that this is the first study to compare detailed body composition parameters between PsA, healthy controls and type 2 diabetes using the gold standard in body composition analysis, MRI. By matching PsA individuals with healthy controls of similar age, sex and BMI, we minimized covariate confounding. Limitations include cross-sectional study design and the modest number of PsA participants. Results should be confirmed in a future study with a larger number of PsA participants including those with varying disease activity and biologic agent use. While IMAPA imaging was conducted in a different centre to UK Biobank, all images were obtained using standardised AMRA protocols and analysed centrally by AMRA. Further, between MR scanner bias and reproducibility has recently been published and has shown that the magnitude of any systematic differences between MR scanners is smaller than the effect sizes observed in this study, lending confidence that the findings we report are genuine and robust [11].

In conclusion, individuals with PsA are metabolically distinct with greater VAT and ectopic liver fat and lower thigh muscle volume than age-, sex- and BMI-matched healthy counterparts, and associated with greater propensity to CHD and type 2 diabetes. Indeed, PsA body fat distribution was more in line with the pattern observed in type 2 diabetes. These novel MRI findings suggest that, as is the case in patients with type 2 diabetes, weight loss should be a core component of PsA disease management to lessen cardiometabolic risk. Large randomized placebo-controlled trials are now needed to prove weight loss improves body composition and outcomes in PsA, as suggested by prior studies [20].

## Supplementary Material

keaa604_Supplementary_Data
