# Supplementary material for: Psoriatic arthritis is associated with adverse body composition predictive of greater coronary heart disease and type 2 diabetes propensity – a cross-sectional study
Source: Rheumatology (Oxford). 2020 Nov 4;60(4):1858–62. doi: 10.1093/rheumatology/keaa604 (PMC8024001; doi:10.1093/rheumatology/keaa604)
Supplement: keaa604_Supplementary_Data [file keaa604_supplementary_data.zip › keaa604-suppl_data/rhe-20-1011-File004.docx]

**SUPPLEMENTARY MATERIAL**

**Supplementary Table S1. Baseline characteristics of psoriatic arthritis (PsA) participants**

| Variable | Median (IQR) or n (%) |
| --- | --- |
| Disease activity |  |
| 68 tender joint count | 8 (5, 16) |
| 66 swollen joint count | 7.5 (4, 14) |
| DAS28-ESR score | 3.91 (3.31, 5.24) |
| PASI | 4.0 (1.4, 6.6) |
| Physician global disease activity | 57 (45, 65) |
| Patient global disease activity | 54.5 (35, 71) |
| HAQ-DI | 0.94 (0.13, 1.42) |
| CRP | 3.5 (2, 16) |
| DMARD use | 6 (24) |
| Cardiometabolic characteristics |  |
| HbA1c (mmol/mol) | 37.5 (36.2, 40.5) |
| Total cholesterol (mmol/L) | 4.9 (4.4, 5.4) |
| Systolic blood pressure | 136 (129, 149) |
| Diastolic blood pressure | 84 (78, 90) |

N=26 PsA participants. Values are median (interquartile range (IQR)) or number (n) (%).

CRP: C-reactive protein; DAS28-ESR: disease activity score (28 joints) erythrocyte sedimentation rate; DMARD: disease-modifying antirheumatic drug; HAQ-DI: health assessment questionnaire disability index; HbA1c: glycated haemoglobin; PASI: Psoriasis Area and Severity Index.

**Supplementary figure legend**

**Supplementary Figure S1. Scatterplot of propensity to CHD and type 2 diabetes for PsA participants (red) compared to UK Biobank (grey).** Values are mean (standard deviation). CHD: coronary heart disease; MDF: metabolic disease free; PsA: psoriatic arthritis.s
