# Supplementary figures and images for: Psoriatic arthritis is associated with adverse body composition predictive of greater coronary heart disease and type 2 diabetes propensity – a cross-sectional study
Source: Rheumatology (Oxford). 2020 Nov 4;60(4):1858–62. doi: 10.1093/rheumatology/keaa604 (PMC8024001; doi:10.1093/rheumatology/keaa604)

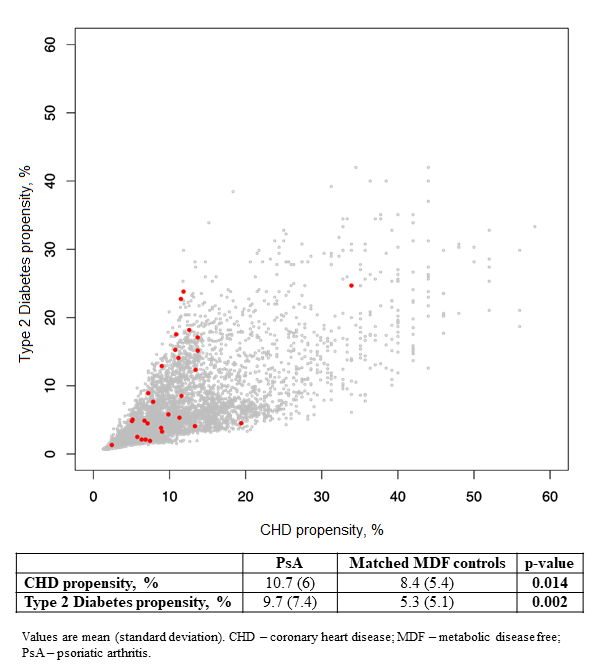

Supplement: keaa604_Supplementary_Data [file keaa604_supplementary_data.zip › keaa604-suppl_data/Suppl fig S1 updated.tif]
